# A Selective Histamine H_4_ Receptor Antagonist, JNJ7777120, Is Protective in a Rat Model of Transient Cerebral Ischemia

**DOI:** 10.3389/fphar.2018.01231

**Published:** 2018-10-29

**Authors:** Ilaria Dettori, Lisa Gaviano, Alessia Melani, Laura Lucarini, Mariaconcetta Durante, Emanuela Masini, Felicita Pedata

**Affiliations:** Department of Neuroscience, Psychology, Drug Research and Child Health (NEUROFARBA), Division of Pharmacology and Toxicology, University of Florence, Florence, Italy

**Keywords:** H_4_ histamine receptor, JNJ7777120, middle cerebral artery occlusion, brain ischemia, neuroinflammation, rats

## Abstract

Cerebral ischemia is a multifactorial pathology characterized by different events evolving in time. The acute injury, characterized by excitoxicity, is followed by a secondary brain injury that develops from hours to days after ischemia. Extracellular levels of histamine increase in the ischemic area after focal cerebral ischemia induced by occlusion of the middle cerebral artery (MCAo). The histamine H_4_ receptor (H_4_R) is predominantly expressed in cell types of immune system where is involved in the regulation of immunological and inflammatory responses, and in numerous area of the Central Nervous System (CNS) including cortex and striatum. Our aim was to assess the putative neuroprotective effects of the potent and selective H_4_R antagonist, JNJ7777120 (JNJ), chronically administered (1 mg/kg, i.p., twice/day for 7 days) on damage parameters in a rat model of focal ischemia induced by transient MCAo (tMCAo). Chronic treatment with the H_4_R antagonist JNJ, significantly protected from the neurological deficit and from body weight loss after tMCAo. Seven days after the ischemic insult, JNJ reduced the volume of the ischemic cortical and striatal damage, the number of activated microglia and astrocytes in the ischemic cortex and striatum and decreased the plasma levels of IL-1β and TNF-α, while increased the levels of IL-10. Two days after ischemia, JNJ has reduced granulocyte infiltration in the ischemic area. Results demonstrate that the selective antagonist of H_4_R, JNJ, systemically and chronically administered after ischemia, reduces the ischemic brain damage, improves the neurological deficit and decreases blood pro-inflammatory cytokines, suggesting that H_4_R is a valuable pharmacological target after focal brain ischemia.

## Introduction

Histamine is a relevant neurotransmitter/neuromodulator in the CNS. Up to now, four subtypes of histamine receptors (HR) have been identified. Three (H_1_–H_3_) of the four histamine receptors that have been characterized are expressed in the brain and on hematopoietic cells with the exception of the H_3_ receptor (H_3_R) which acts exclusively in the CNS as a presynaptic auto-receptor that inhibits the release of histamine from neurons (Martinez-Mir et al., 1990). In the past decades, the histaminergic system has been implicated in the modulation of several brain disorders, but the role of histamine in ischemic damage has not yet been fully defined. Histamine receptors could play important roles in cerebral ischemia by acting on multiple targets (Adachi, 2005; Hu and Chen, 2012). The expression and the binding density of central histamine H_1_ receptor (H_1_R) and H_3_R increase while that of H_2_ receptor (H_2_R) decrease in the basal ganglia after cerebral ischemia (Lozada et al., 2005). Moreover, evidence has shown that histamine administered intracerebroventricularly (i.c.v.) exerts a protective effect on neurological injury after transient forebrain ischemia in gerbil (Fujitani et al., 1996) or after MCAo in the rat (Hamami et al., 2004). In the rat MCAo model, histamine (Adachi et al., 2005; Hu and Chen, 2012) and its precursor histidine (Fang et al., 2014) exert a protective effect by reducing excitoxicity in the early phase of ischemia acting on H_1_R and H_2_R. On the contrary, the activation of central H_3_R aggravates delayed neuronal death after ischemia induced by 4-vessel occlusion in the rat (Adachi et al., 1993). Consistently, damage induced by tMCAo in the mouse is attenuated by H_3_R antagonists and in H_3_R knockout mice (Yan et al., 2014).

After the early excitoxicity, brain ischemia is characterized by a strong inflammatory response which further contributes to the progression of injury damaging neurons and glia by releasing proteolytic enzymes, reactive oxygen species, and cytokines (White et al., 2000). Consistent results characterize the most recently discovered member of the G protein-coupled receptor subfamily of histamine, the H_4_R (Nakamura et al., 2000; Oda et al., 2000; Liu et al., 2001; Nguyen et al., 2001), as the main immune system histamine receptor with a pro-inflammatory role (Jutel et al., 2009; Leurs et al., 2009). H_4_R is mainly expressed on hematopoietic cells (Zampeli and Tiligada, 2009) of the immune system, including mast cells, leukocytes, dendritic cells, and T lymphocytes (Thurmond et al., 2008; Jutel et al., 2009; Leurs et al., 2009; Gutzmer et al., 2011). However, its role in brain immune responses was scarcely studied. To date there is no information on the role of H_4_R in brain ischemia. The aim of our study was to investigate such a role by the use of the potent and selective H_4_R antagonist, JNJ, in the rat model of transient focal ischemia.

## Materials and Methods

### General Description

This is a randomized preclinical trial and blinding was implemented only for treatment and for outcome evaluation (neuroscore and histology).

### Animals

Male Wistar rats (Envigo, Italy) weighting 270–290 g were used. Animals were housed in groups of three with free access to food and water and kept under standardized temperature, humidity, and light conditions (12 h light/dark cycle) within the animal house facility of the University of Florence. The experimental procedures described were approved by the local Animal Welfare Body (AWB) of the University of Florence and authorized by the Italian Ministry of Health (Authorization n. 118/2016-PR). The ethical policy of the University of Florence complies with to the Directive 2010/63/EU of the European Parliament and to the Italian Regulation DL 26/2014 on the protection of animals used for scientific purposes. According to the law, all efforts were made to fulfill to the principle of 3Rs.

### Surgery

Focal cerebral ischemia was induced by intraluminal MCAo in the right hemisphere. The animals were anesthetized with 5.0% isoflurane (Baxter International) and spontaneously inhaled 1.0–2.0% isoflurane in air by the use of a mask. Body core temperature was maintained at 37°C with a recirculating pad and K module and was monitored via an intrarectal type T thermocouple (Harvard, Kent, United Kingdom). The surgical procedure to occlude the MCA consisted in insertion of a 4-0 nylon monofilament (Doccol corporation, United States), via the external carotid artery into the internal carotid artery in order to block the origin of the MCA according to the procedure described by Melani et al. (1999)_._ One hour after occlusion, animals were re-anesthetized with isoflurane and brain tissue reperfused by withdrawing the filament. The sham operation was conducted by inserting the filament into the internal carotid artery and immediately withdrawing it.

### Inclusion Criteria

Circling behavior after awakening from anesthesia during MCAo + mNSS score >6 at 24 h after MCAo.

### Exclusion Criteria

No ischemic lesion at histology.

Major protocol violation (i.e., errors in ischemia time).

### Drug Administration and Experimental Groups

The H_4_R antagonist 1-[(5-chloro-1H-indol-2yl)carbonyl]-4methylpiperazine, (JNJ7777120; Johnson & Johnson, San Diego, CA, United States) was dissolved in saline with 1.1% dimethyl sulfoxide (DMSO). The JNJ was chronically administered at the dose of 1 mg/kg, intraperitoneal (i.p.).

A total of 50 rats were operated, 10 animals died: four during operation and six animals died 24 h after operation, two JNJ-treated rats and four vehicle-treated rats. No significant adverse effects were observed in JNJ-treated animals.

Animals subjected to tMCAo were sacrificed 7 days after ischemia. Rats were randomly allocated in the following groups: (1) sham-operated rats (*n* = 6): did not receive any treatment; (2) tMCAo + vehicle group (*n* = 13): saline with DMSO (1.1%) administered (i.p.) twice/day for 7 days, starting 4 h after tMCAo; (3) tMCAo + JNJ group (*n* = 10): JNJ administered (i.p.) twice/day for 7 days, starting 4 h after tMCAo. Blood for cytokines assay was collected in three sham-operated animals.

A group of animals subjected to tMCAo was sacrificed 2 days after ischemia. Rats were randomly allocated in the following groups: (1) Sham-operated rats (*n* = 3): did not receive any treatment; (2) tMCAo + vehicle group (*n* = 4): saline with DMSO (1.1%) administered (i.p.) twice/day for 2 days, starting 4 h after tMCAo; (3) tMCAo + JNJ group (*n* = 4): JNJ administered (i.p.) twice/day for 2 days starting 4 h after tMCAo.

### Neurological Deficit

The neurological deficit was evaluated by modified Neurological Severity Score (mNSS) test described by Chen et al. (2001). All tests were carried out before tMCAo and 1, 5, and 7 days after tMCAo. The mNSS test evaluates the sensorimotor deficit composed of motor, sensory, reflex, and balance tests. The test is graded on a scale from 0 (normal score) to 18 (maximal deficit score). Beam balance test score affects 1/3 of the total mNSS score.

### Body Weight Evaluation

Rats used to induce cerebral ischemia were in the body weight ranging from 270 to 290 g. The body weight was evaluated before tMCAo and after 1, 5, and 7 days from occlusion. The weight variation after ischemia of each animal was evaluated with respect to its own pre-ischemia weight.

### Ischemic Brain Damage

Rats were anesthetized with Zoletil 50/50 (100 mg/kg i.p., Virbac, Carros, Francia) and were perfused transcardially with an ice-cold 4% paraformaldehyde solution (in phosphate buffer, pH 7.4). Brains were post-fixed overnight and cryoprotected in a 18% sucrose solution (in phosphate buffer) for at least 48 h. Brains were cut with a cryostat and coronal sections (20 μm) were collected at 200 μm intervals at eight different levels through the striatum (from +2.2 mm to -1.0 mm from Bregma) (König and Klippel, 1967). Seven days after tMCAo, brain slices were stained by acetate cresyl violet (1%) or by hematoxylin and eosin (H&E). Histological analysis by cresyl violet staining allows to clearly define the infarct area and volume up to 1 week after ischemia (Rousselet et al., 2012). To evaluate area and volume of ischemic damage, eight cresyl violet-stained brain sections per animal were placed directly on the scanning screen of a color flatbed scanner (CanoScan LiDE 90; Canon). Following image acquisition, the images were analyzed using ImageJ software. The measurements of infarct area in striatum and cortex were obtained by manually outlining the margins of infarcted area. Ischemic cortical and striatal volumes were calculated by multiplying the infarcted area by the slice thickness and summing the volume of the eight slices.

After H&E staining, heterochromatic nuclei were counted at Bregma level within an optical field at 40 × in ischemic cortex and striatum. Data were then averaged and expressed as mean ± SEM of number cells per optical field of “*n*” animals.

### Blood Infiltration and Gliosis

Immunohistochemistry studies were performed with avidin–biotin complex techniques. Primary antibodies were mouse monoclonal antibody, anti-HIS-48 (specific for granulocytes; 1:50, Santa Cruz Biotechnology), mouse monoclonal antibody, anti-GFAP (specific for astrocytes; 1:300, BD Transduction Laboratories) and rabbit polyclonal antibody, anti-IBA-1 (specific for microglia; 1:300, Wako Chemicals; see Melani et al., 2014). All sections were examined using an Olympus BX40 microscope (Olympus, Milan, Italy) and photographed using a digital camera (Olympus DP50). To evaluate the number of granulocytes, astrocytes, and microglia, cells were counted within an optical field at 40 × in ischemic cortex and striatum. HIS-48-positive cells were counted at seven different levels per animal (+2.0 mm to -1.0 mm from the Bregma). GFAP- and IBA-1-positive cells were counted at Bregma level. Data were then averaged and expressed as mean ± SEM of number cells per optical field of “*n*” animals.

### Determination of TNF-α, IL-1β, and IL-10

The levels of interleukine-1β (IL-1β) and Tumor Necrosis Factor-α (TNF-α) pro-inflammatory cytokines and of interleukine-10 (IL-10) a regulatory cytokine, were measured on aliquots (200 μl) of plasma from sham-operated rats (*n* = 3), vehicle-treated rats (*n* = 5) and JNJ-treated rats (*n* = 5), using commercial ELISA kits (ELISA Ready-Set-Go, eBioscience, San Diego, CA, United States), following the protocol provided by the manufacturer (Lucarini et al., 2016). Results are expressed as pg of protein/ml of plasma.

### Statistical Analysis

Data were statistically analyzed by one-way analysis of variance (ANOVA) followed by Newman–Keuls multiple comparison test, Repeated Measures two-way analysis of variance (ANOVA) followed by Tukey *post hoc* test and by unpaired Student’s *t* test as specified in text and in figure legends. A value of *p* < 0.05 was considered statistically significant. The statistical analysis was performed utilizing GraphPad Prism7.

## Results

### The Histamine H_4_ Receptor Antagonist Reduces the Neurological Deficit

**FIGURE 1:**
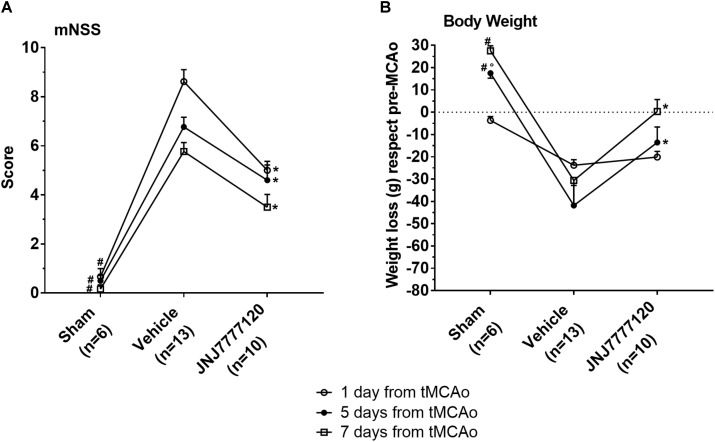
Effect of chronic treatment with JNJ (1 mg/kg i.p.) on neurological deficit **(A)** and body weight loss **(B)**. Data are expressed as mean ± SEM of “*n*” rats. **(A)** mNSS test: the score is evaluated before and after 1, 5, and 7 days from tMCAo in each rat group. Repeated Measures two-way ANOVA followed by Tukey *post hoc* test: ^#^*p* < 0.0001 sham-operated vs. chronic JNJ- and vehicle-treated rats; ^∗^*p* < 0.001–0.0001 chronic JNJ-treated vs. vehicle-treated rats. **(B)** Body weight loss: sham-operated rats increase their body weight in the period after the operation. The body weight loss of tMCAo rats was calculated as the mean ± SEM of the difference between body weight at each time point and pre-operation body weight. Repeated Measures Two-way ANOVA followed by Tukey *post hoc* test: ^#^*p* < 0.0001 sham-operated vs. vehicle-treated rats; °*p* < 0.025 sham-operated vs. chronic JNJ-treated rats; ^∗^*p* < 0.004–0.01 chronic JNJ-treated vs. vehicle-treated rats.

The mNSS was performed according to Chen et al. (2001) before tMCAo and 1, 5, and 7 days after tMCAo. The test showed that sham-operated rats had a neurological score of 0.2–0.7 in the period from 1 to 7 days after tMCAo (Figure 1A). Twenty-four hours after tMCAo, vehicle-treated rats showed a clear neurological deficit with a neurological score of 8.6 ± 0.5 (mean ± SEM) that defines a moderate injury. The neurological impairment spontaneously recovered up to 7 days after tMCAo when it was reduced to 5.8 ± 0.4 that represents a mild injury. Chronic treatment with the H_4_R antagonist, JNJ, at the dose of 1 mg/kg, significantly reduced the neurological deficit at each time point up to 7 days after tMCAo. Repeated Measures Two-way ANOVA calculated for the two factors: treatment and time after tMCAo, showed that treatment factor (*F*_2,26_ = 82.35; *p* < 0.0001), time factor (*F*_2,52_ = 12.29; *p* < 0.0001) and interaction between treatment and time (*F*_4,52_ = 2.66; *p* < 0.042) were statistically significant. The Tukey *post hoc* test indicated that sham-operated rats had a neurological score significantly different from vehicle-treated and JNJ-treated rats at each time point (1, 5, and 7 days after tMCAo, *p* < 0.001). The chronic treatment with JNJ significantly reduced the neurological deficit with respect to vehicle-treated rats at 1, 5, and 7 days (*p* < 0.001–0.0001) after tMCAo.

### The Histamine H_4_ Receptor Antagonist Reduces Body Weight Loss After tMCAo

Male Wistar rats used in this study had a body weight in the range of 270–290 g. Twenty-four hours after operation, sham-operated rats lost 3.7 ± 0.7 g weight, then they increased in body weight as evaluated up to 7 days after tMCAo. Vehicle-treated rats lost 23.8 ± 2.5 g 1 day after tMCAo, 41.9 ± 9.0 g 5 days and 30.6 ± 10.8 g 7 days after tMCAo (Figure 1B). Chronic treatment with JNJ significantly reduced the body weight loss at 5 and 7 days after tMCAo with respect to vehicle-treated rats. Repeated Measures two-way ANOVA, calculated for the two factors: treatment and time after tMCAo, showed that treatment factor (*F*_2,26_ = 11.87; *p* < 0.0002), time factor (*F*_2,52_ = 7.95; *p* < 0.001) and interaction between treatment and time (*F*_4,52_ = 6.08; *p* < 0.0004), were statistically significant. The Tukey *post hoc* test indicated that in sham-operated rats, body weight was different with respect to vehicle-treated rats 5 and 7 days after tMCAo (*p* < 0.0001) and to JNJ-treated rats at 5 days after tMCAo (*p* < 0.025). The chronic treatment with JNJ significantly decreased body weight loss at 5 days (of 67.5%, *p* < 0.01) and at 7 days (of 99.0%; *p* < 0.004) after tMCAo, with respect to vehicle-treated rats.

### The Histamine H_4_ Receptor Antagonist Reduces Brain Ischemic Damage After tMCAo

**FIGURE 2:**
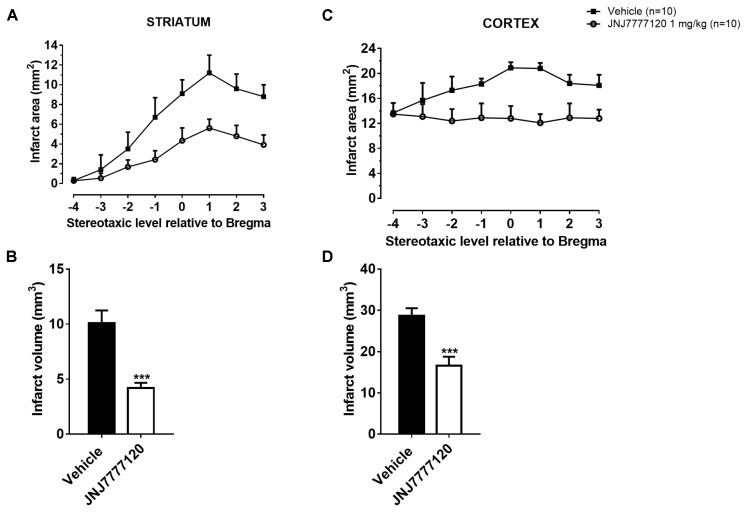
Effect of chronic treatment with JNJ (1 mg/kg i.p.) on infarct area **(A,C)** and infarct volume **(B,D)** in the striatum and cortex 7 days after tMCAo. Data are the mean ± SEM of infarct area measured at eight predetermined coronal levels through the brain of “*n*” rats. Bregma = 0 (König and Klippel, 1967). Bar graphs show the infarct volume calculated as mean ± SEM in the striatum and cortex. Unpaired Student’s *t*-test: ^∗∗∗^*p* < 0.0001–0.0005 vs. vehicle-treated rats.

**FIGURE 3:**
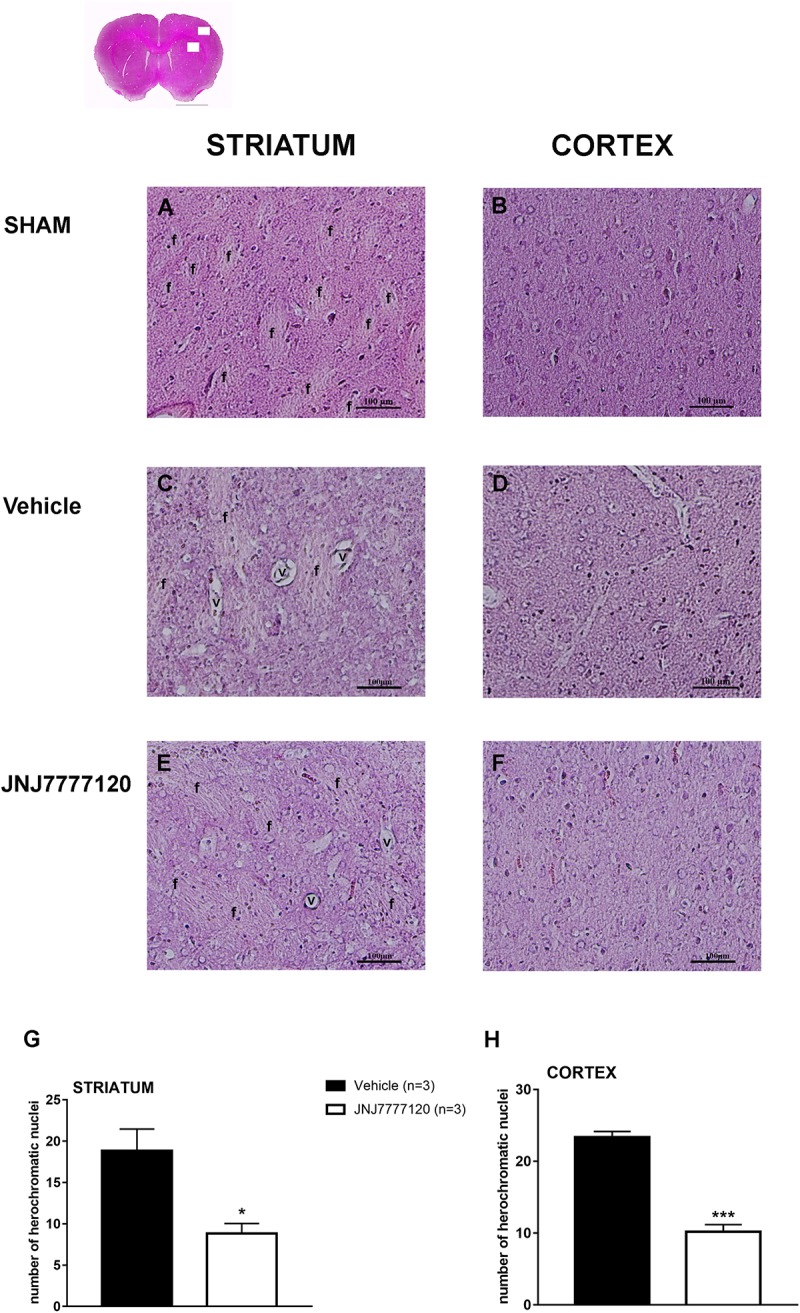
Effect of chronic treatment with JNJ (1 mg/kg i.p.) on cytoarchitecture of the ischemic striatum and cortex after tMCAo. Upper part: representative photomicrograph of a histological section of control rat (at Bregma + 1.5 mm) (König and Klippel, 1967). The two white boxes indicate the ischemic striatal and cortical H&E stained area. Scale bar = 2 mm. Sham-operated rats: **(A)** in the dorsal striatum, the typical caudate-putamen cytoarchitecture is appreciable, numerous transversally sectioned white matter *fascicula* (*f*) are surrounded by gray matter containing diverse type of neurons distinct on the basis of their size and shape (Danner and Pfister, 1981);**(B)** in the fronto-parietal cortex the typical columnar organization is appreciable. Vehicle-treated ischemic rats: **(C)** in most part of the ischemic dorsal striatum the cytoarchitecture is lost, numerous and dilated vessels (v) and small and heterochromatic nuclei are present;**(D)** in the ischemic fronto-parietal cortex the columnar organization is not appreciable, the interstitial spaces are enlarged, vessels are numerous and dilated and numerous heterochromatic small nuclei are present. JNJ-treated ischemic rats: note the recovery of the staining intensity and the decrease of heterochromatic small nuclei; **(E)** in the ischemic dorsal striatum the cytoarchitecture of the white matter *fascicula* is again recognizable; **(F)** in the ischemic fronto-parietal cortex the columnar organization is appreciable. Scale bar = 100 μm. Bar graphs represent the mean ± SEM of the number of heterochromatic nuclei per optical field (40 ×) present at Bregma = 0 coronal level of three rats, in ischemic striatum **(G)** and in ischemic cortex **(H)**. Unpaired Student’s *t*-test: ^∗^*p* < 0.02; ^∗∗∗^*p* < 0.0005 vs. vehicle rats.

Figure 2 shows the extent of ischemic damage evaluated as infarct area (Figures 2A,C) and infarct volume (Figures 2B,D) in ischemic striatum and cortex of vehicle- and JNJ-treated rats 7 days after tMCAo. Chronic treatment with JNJ significantly reduced the infarct in both area. The striatal and cortical infarct volumes were reduced by 58.4% and 42.2%, respectively. (Unpaired Student’s *t*-test: *p* < 0.0001–0.0005). Sham-operated rats did not show any damage. To characterize the cytoarchitecture of the ischemic cortex and striatum 7 days after tMCAo, ischemic tissue was stained by H&E (Figure 3). Seven days after transient ischemia, H&E staining showed a decrease in staining intensity in vehicle-treated rats (Figures 3C,D) compared to sham-operated rats (Figures 3A,B). Parenchyma showed numerous small and heterochromatic nuclei both in ischemic cortex and striatum. The typical cytoarchitecture of these two regions (for a description see Danner and Pfister, 1981) was lost. In the striatum, the white matter *fascicula* (f) were no more recognizable; in the fronto-parietal cortex the columnar organization was not appreciable. Figures 3E,F showed that chronic administration of JNJ was associated with a recovery of the staining intensity and with a reduction of heterochromatic small nuclei staining in both brain regions. The cytoarchitecture of the white matter *fascicula* was recognizable in the dorsal corpus striatum (Figure 3E) and the columnar organization was appreciable in the fronto-parietal cortex (Figure 3F). Quantitative analysis showed that chronic JNJ treatment significantly reduced heterochromatic nuclei number in the ischemic striatum (*p* < 0.02; Figure 3G) and in the ischemic cortex (*p* < 0.0005; Figure 3H).

### Effect of Treatment With Histamine H_4_ Receptor Antagonist on Gliosis After tMCAo

**FIGURE 4:**
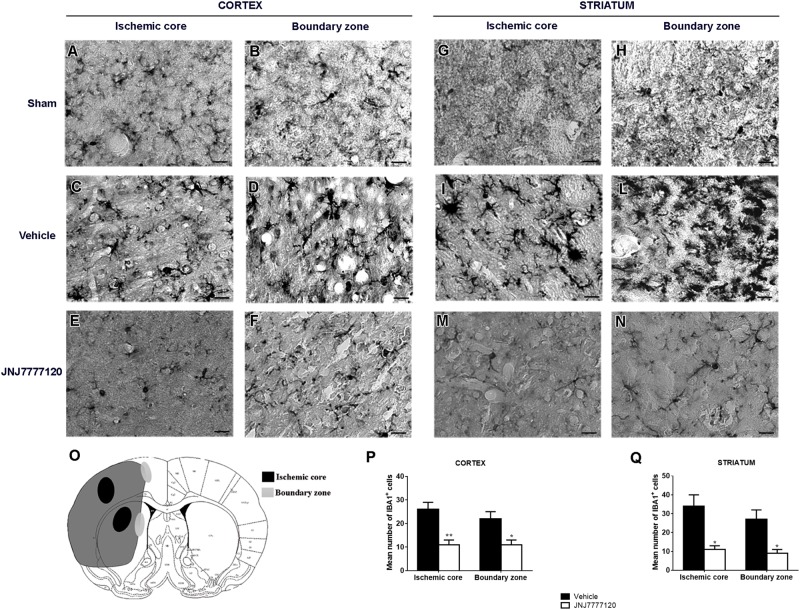
Effect of chronic treatment with JNJ (1 mg/kg i.p.) on microgliosis in the ischemic core and boundary zone 7 days after tMCAo. Schematic brain picture **(O)** indicates regions where photomicrographs of ischemic core and boundary zone were captured. In the sham-operated rats, microglia (black cells) has morphological features of resting cells with small cell body and faintly stained thin processes **(A,B–G,H)**. Seven days after ischemia, an increase of IBA-1 immunostaining intensity is observed both in ischemic cortex and striatum. In the cortical and striatal ischemic core, microglia has a round macrophage-like morphology, typical of activated cells **(C,I)**. In the cortical and striatal boundary zones, IBA-1 positive cells have a round cell body with short and thick processes, a morphology defined reactive microglia **(D,L)** (Cerbai et al., 2012). Treatment with JNJ reduces IBA-1 immunostaining intensity and hypertrophic features in the core and in the boundary zone of both cortex **(E,F)** and striatum **(M,N)**. Scale bar = 20 μm. Bar graphs represent the mean ± SEM of the number of IBA1^+^ cells, per optical field (40 ×), present at Bregma = 0 coronal level of four rats, in the ischemic core and boundary zone of both cortex **(P)** and striatum **(Q)**. Unpaired Student’s *t*-test: ^∗^*p* < 0.01–0.02; ^∗∗^*p* < 0.005 vs. vehicle rats.

Seven days after surgery, microglia, and astrocytes were characterized by IBA-1 and GFAP immunostaining, respectively. In Figure 4O the regions of ischemic core and boundary zone were indicated. In sham-operated rats, resting microglia (Figures 4A,B,G,H) was diffusely distributed throughout the cortex and striatum. Microglia had morphological features typical of resting cells with a small cell body and faintly stained thin processes. Seven days after tMCAo, a definite increase of IBA-1 immunostaining was observed both in striatum and cortex. In the cortical and striatal core, microglia assumed a round macrophage-like morphology, typical of activated cells (Figures 4C,I); in the boundary zone, microglial cells showed a large cell body with thick and short processes (Figures 4D,L), an intermediate morphology between resting and activated microglia defined reactive microglia (Melani et al., 2006; Cerbai et al., 2012). Chronic treatment with JNJ reduced IBA-1 immunostaining intensity and hypertrophic features in the core and in the boundary zone of both cortex (Figures 4E,F) and striatum (Figures 4M,N). Quantitative analysis (Figures 4P,Q) showed that JNJ treatment significantly reduced IBA-1^+^ cell number in the cortical ischemic core (*p* < 0.005) and in the boundary zone (*p* < 0.02; Figure 4P) and in the striatal ischemic core (*p* < 0.01; Figure 4Q) and in the boundary zone (*p* < 0.01; Figure 4O).

**FIGURE 5:**
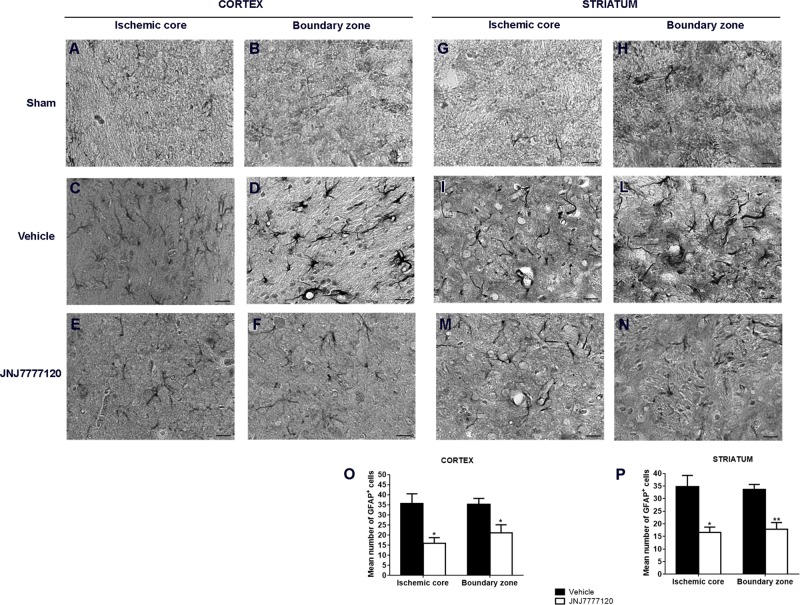
Effect of chronic treatment with JNJ (1 mg/kg i.p.) on astrogliosis in the ischemic core and boundary zone 7 days after tMCAo. In the sham-operated rats, astrocytes (black cells) have morphological features of resting cells with small cell body and thin ramified processes both in cortex **(A,B)** and in the striatum **(G,H)**. Seven days after ischemia, an increase of GFAP immunostaining intensity is observed both in ischemic cortex and striatum. In the cortical and striatal ischemic core, astrocytes have morphological features of suffering cells **(C,I)**. In the cortical and striatal boundary zones, GFAP-positive cells show a highly hypertrophic cell body and long and thick processes **(D,L)**. Treatment with JNJ reduces GFAP immunostaining intensity and hypertrophic features in the core and in the boundary zone of both cortex **(E,F)** and striatum **(M,N)**. Scale bar = 20 μm. Bar graphs represent the mean ± S.E.M. of the number of GFAP^+^ cells per optical field (40 x), present at Bregma = 0 coronal level of four rats, in the ischemic core and boundary zone of both cortex **(O)** and striatum **(P)**. Unpaired Student’s *t*-test: ^∗^*p* < 0.01–0.02; ^∗∗^*p* < 0.001 vs. vehicle rats.

In sham-operated rats, resting astrocytes (Figures 5A,B,G,H) were diffusely distributed throughout the cortex and striatum. Astrocytes had morphological features typical of resting cells with a small cell body and faintly stained thin processes. Seven days after tMCAo, a definite increase of GFAP immunostaining was observed both in striatum and cortex. In the ischemic core, astrocytes showed a smaller cell body and shorter processes (Figures 5C,I) while in the boundary zone, they showed a highly hypertrophic cell body and long and thick processes (Figures 5D,L). Chronic treatment with JNJ reduced the GFAP immunostaining intensity both in core and boundary zone and astrocytes appeared less hypertrophic (Figures 5E,F,M,N). Quantitative analysis (Figures 5O,P) showed that JNJ treatment significantly reduced GFAP^+^ cell number in cortical ischemic core (*p* < 0.02; Figure 5O) and boundary zone (*p* < 0.02; Figure 5O) and in the striatal ischemic core (*p* < 0.01) and boundary zone (*p* < 0.001; Figure 5P).

### Effect of Treatment With the Histamine H_4_ Receptor Antagonist on Cytokine Plasma Levels After tMCAo

**FIGURE 6:**
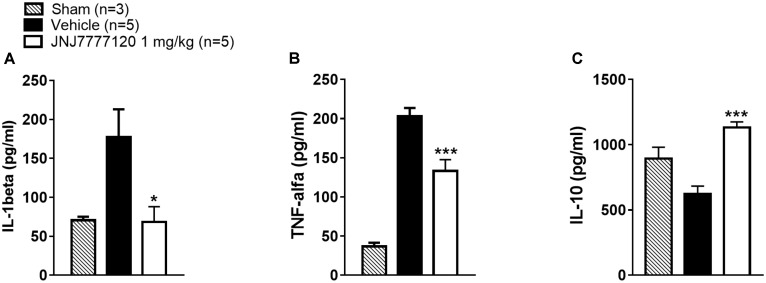
Effect of chronic treatment with JNJ (1 mg/kg i.p.) on IL-1β **(A)**, TNF-α **(B)**, and IL-10 **(C)** plasma levels. Results are expressed as pg of protein/ml of plasma and values are mean ± SEM. One-way ANOVA: ^∗∗∗^*p* < 0.0005; ^∗^*p* < 0.02 vs. vehicle rats.

Seven days after tMCAo, plasma levels of pro-inflammatory cytokines IL-1β and TNF-α were significantly increased while the plasma level of IL-10, a regulatory cytokine with an anti-inflammatory action was reduced in vehicle-treated rats as compared to sham-operated rats (Figures 6A–C). Chronic treatment with JNJ significantly reduced IL-1β and TNF-α levels while restored the plasma level of IL-10 (One-way ANOVA: *p* < 0.0005–0.02) (Figures 6A–C).

### Effect of Treatment With the Histamine H_4_ Receptor Antagonist on Blood Cell Infiltration After tMCAo

**FIGURE 7:**
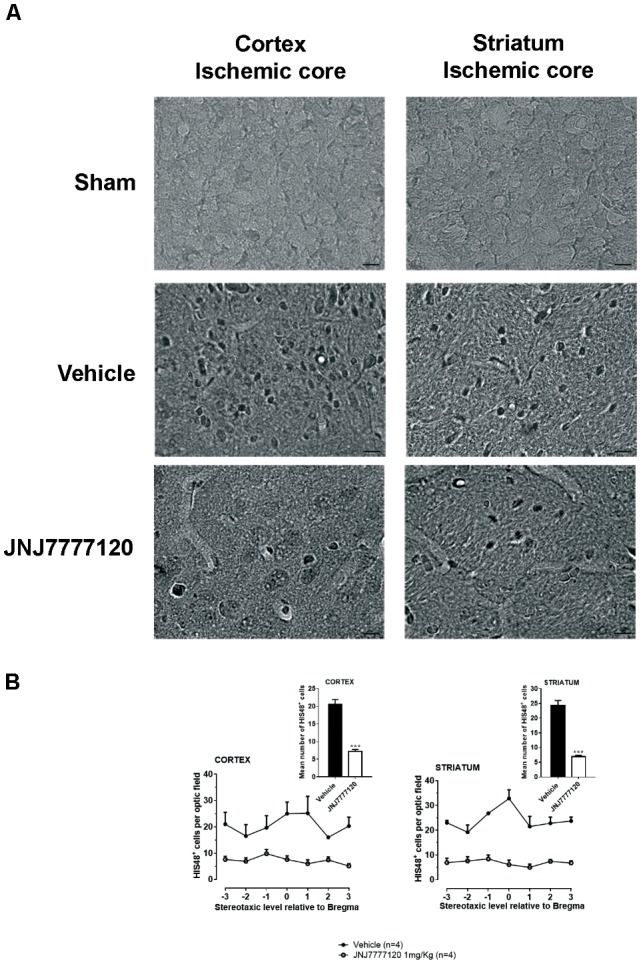
Effect of chronic treatment with JNJ (1 mg/kg i.p.) on blood cell infiltration in the ischemic core 2 days after tMCAo. **(A)** Representative micrographs of HIS-48-positive cells detected in cortical and striatal ischemic core obtained by optical microscopy at 60 × of magnification. Scale bar = 10 μm. JNJ reduces HIS-48-positive cells in cortex and striatum. **(B)** Data are mean ± SEM of the number of HIS-48-positive cells per optical field (40 ×) counted in seven coronal levels through the brain of “*n*” rats taking Bregma = 0 (König and Klippel, 1967). Unpaired Student’s *t*-test:^∗∗∗^*p* < 0.0001–0.0002.

Two days after tMCAo, HIS-48^+^ cells (granulocytes) were detected in the cortical and striatal ischemic core (Figure 7A). Seven days after tMCAo, granulocytes were anymore detectable in the ischemic tissue (data not shown).

Chronic treatment with JNJ significantly reduced the number of HIS-48^+^ cells both in the cortical ischemic core (mean ± SEM: 20.5 ± 1.4 cells/optical field in vehicle- vs. 7.2 ± 0.5 cells/optical field in JNJ-treated rats; unpaired Student’s *t*-test: *p* < 0.0002) and in the striatal ischemic core (mean ± S.E.M: 24.3 ± 1.7 cells/optical field in vehicle- vs. 6.9 ± 0.4 cells/optical field in JNJ-treated rats; unpaired Student’s *t*-test: *p* < 0.0001) (Figure 7B).

## Discussion

Results demonstrate that the selective antagonist of H_4_R, JNJ, systemically and chronically administered after ischemia, reduces the ischemic brain damage and improves the neurological deficit.

In the experimental model of focal cerebral ischemia induced by MCAo in rats, the levels of histamine and tele-methylhistamine evaluated by microdialysis in the striatum and cerebral cortex gradually increase reaching levels threefold and twofold higher, respectively, than those of the contralateral side and become statistically significant 6–12 h after induction of ischemia (Adachi et al., 1991). JNJ was described as a lipophilic drug that readily crosses Blood Brain Barrier (BBB) (Funke et al., 2013) and H_4_R recently identified in cells of CNS, can account for protective effects of the H_4_ antagonist in MCAo rats. In the human brain, H_4_R mRNA was detected by real-time PCR (RT-PCR) in brain regions including hippocampus, cortex, thalamus, and amygdala and in the rat brain in the cortex, cerebellum, brainstem, amygdala, thalamus, and striatum (Strakhova et al., 2009). As to the cellular localization, expression of H_4_R mRNA was detected by immunohistochemistry in neurons of several regions of human and mouse brain, such as thalamus, hippocampus, and cerebral cortex (Connelly et al., 2009). Although the most accurate qPCR technique has revealed only low levels of H_4_R mRNA in human brain and criticism was raised concerning the consistency of literature data that demonstrate the presence of H_4_R in the brain (Schneider and Seifert, 2016), the brain H_4_R is functional. Indeed, in the somatosensory cortex, the H_4_R agonist 4-methyl histamine directly hyperpolarizes neurons and promotes outwardly rectifying currents (Connelly et al., 2009). Acutely after ischemia, glutamate acting on “non-NMDA” and NMDA receptors acts as the main promoter of excitoxicity that brings to glial reactivity and neuron death (Chamorro et al., 2016). However, the H_4_R hyperpolarization effect together with the observation that H_4_R did not act as hetero-receptors on thalamocortical glutamatergic terminals (Connelly et al., 2009) do not support the notion that the protective effects of JNJ are attributable to reduced release of glutamate from neurons.

In addition to neurons, H_4_R has been localized by RT-PCR analysis, immunocytochemistry and western blotting on microglial cells (Ferreira et al., 2012). Here, we report that JNJ chronically administered after ischemia, clearly hampers glial activation reducing both reactivity and number of microglia and astrocytes in the cortical and striatal ischemic core and boundary zones. Consistently, a selective histamine H_4_R antagonist inhibits the histamine induced TNF-α and IL-6 production by primary cultured microglia (Dong et al., 2014) supporting that protective effect of JNJ is attributable to direct antagonism of H_4_R located on rat microglial cells. So far, there is no evidence for the presence of H_4_R on astrocytes. After brain ischemia, the primary acute lethal mechanism of excitoxicity induces the activation of resident immune cells especially microglia and production of inflammation mediators that triggers the so-called “secondary damage” characterized by endothelial expression of adhesion molecules and leukocyte infiltration that on their turn exacerbate neuroinflammation (Iadecola and Anrather, 2011; Pedata et al., 2016). Consistent part of the increase of brain histamine after ischemia (Adachi et al., 1991) might by accounted for by release from mast cells. In a model of transient cerebral ischemia induced by four-vessel occlusion, an increase of mast cells degranulation in association with an increase of histamine content was demonstrated 7 days after ischemia in the adult rat (Hu et al., 2004). Moreover, mast cells were found involved in promoting BBB breakdown and neutrophil infiltration in a mouse model of focal ischemia (McKittrick et al., 2015). Observation that 2 days after tMCAo the H_4_R antagonist has significantly reduced granulocyte infiltration, supports that the histamine H_4_R is involved in promoting BBB breakdown and neutrophil infiltration.

Mast cells residing in the brain are located on or near the cerebrovasculature and, among others mediators, synthetize TNF-α and various eicosanoids as well as pro-inflammatory cytokines that concur to neurovascular inflammation (Hu and Chen, 2012).

Administration of the H_4_R antagonist, JNJ, reduced TNF-α and IL-1β assayed in the peripheral plasma 7 days after ischemia. Such reduction might reflect lower levels of pro-inflammatory cytokines produced in the brain and reaching peripheral blood. It might be also envisaged a peripheral mechanism of action of JNJ. Stroke and inflammation are strictly interrelated. Single-photon emission computed tomography (SPECT) imaging in mice subjected to tMCAo, has revealed that systemic inflammation preceding experimental stroke is associated with markedly augmented brain inflammation and impaired neurologic outcome, but conversely also that brain ischemia induces profound inflammatory changes in the periphery (Szigeti et al., 2015). Inflammatory changes in peripheral organs (especially the lungs and the gut) as early as 2 h after tMCAo in mice has been detected by whole-body SPECT-based imaging protocols (Szigeti et al., 2015). Such peripheral inflammatory changes on their turn might contribute to a worse recovery after stroke. Precise cellular-molecular mechanisms underlying are unclear, but likely reflect a vicious circle responsible of inflammatory mediator production and BBB function. JNJ could reduce TNF-α and IL-1β and increase IL-10 production by antagonizing H_4_R on dendritic cells (Simon et al., 2011) that are activated by histamine that in turn is released by basophiles or perivascular mast cells secondarily to central inflammatory mediators released in the blood after ischemia.

Overall our results stress the key research questions of the predictive value of blood biomarkers in stroke and suggest that JNJ by controlling a secondary inflammatory damage after brain ischemia represents a new interesting target after brain ischemia.

## Author Contributions

FP and EM designed the research. ID, LG, AM, MD, and LL performed the experiments. ID, LG, MD, and LL analyzed the data. ID, LG, and FP interpreted the results and the experiments. ID, LG, MD, and LL prepared the figures. ID, FP, and EM drafted the manuscript. ID, LG, FP, and EM edited and revised the manuscript. All authors read and approved the final version of the manuscript.

## Conflict of Interest Statement

The authors declare that the research was conducted in the absence of any commercial or financial relationships that could be construed as a potential conflict of interest.
